# ANALYTIC METHODS FOR HEALTH DISPARITIES IN ALZHEIMER’S DISEASE AND RELATED DEMENTIA

**DOI:** 10.1093/geroni/igae098.3289

**Published:** 2024-12-31

**Authors:** Igor Akushevich, Arseniy Yashkin, Julia Kravchenko

**Affiliations:** Duke University, Durham, North Carolina, United States; Duke University, Durham, North Carolina, United States; Duke University School of Medicine, Durham, North Carolina, United States

## Abstract

Analysis of Medicare data reveals persistent health disparities related to Alzheimer’s disease and related dementias (AD/ADRD) in the United States. These disparities were measured using empirically estimated age-adjusted rates or hazard ratios (HRs) obtained from proportional hazard (PH) models. However, methods for explaining disparities based on comparisons of HR derived from different PH models have strong limitations and observed differences in HRs cannot be causally interpreted. To overcome these limitations, we developed and applied a series of approaches utilizing both traditional workflows as well as novel methodologies such as the Powers-Yun approach, approaches for population attributable fractions, rank-and-replace methods, and parametric g-formula modeling. We sequentially applied these approaches to two multi-racial cohorts of Medicare beneficiaries with baseline ages 67 and 75 and 10 and 15 years of follow-up, respectively. The estimates of the effects of factors contributing to racial disparities in AD risk showed that social determinants of health were an important predictor of disparity with strong competing effects from risk-related diseases, such arterial hypertension and diabetes mellitus. Traditional methods proved to be useful for univariable and bivariate comparisons but did not allow for clear conclusions on multiple inter-related factors. Estimates obtained through alternative methods while generally consistent were not identical. The Powers-Yun approach and approaches for population attributable fractions demonstrated the most promising results. The parametric g-formula showed great potential for establishing causality. However, further analytic work has to be completed to understand how specific assumptions associated with specific methods impact the estimates.

